# Serological tests for *gambiense* human African trypanosomiasis detect antibodies in cattle

**DOI:** 10.1186/s13071-017-2487-8

**Published:** 2017-11-03

**Authors:** Enock Matovu, Annah Kitibwa, Albert Picado, Sylvain Biéler, Paul R. Bessell, Joseph Mathu Ndung’u

**Affiliations:** 1 College of Veterinary Medicine, Animal Resources and Biosecurity (COVAB), Makerere University, Kampala, Uganda; 2 Foundation for Innovative New Diagnostics (FIND), Campus Biotech, Chemin des Mines, Geneva, Switzerland; 3 Epi Interventions Ltd., Edinburgh, EH3 5HT UK

**Keywords:** Serological tests, Trypanosomes, Specificity, Variant surface glycoproteins, Invariant surface glycoproteins

## Abstract

**Background:**

Serological tests for *gambiense* human African trypanosomiasis (gHAT) detect antibodies to antigens on the cell surface of bloodstream trypanosomes. As trypanosomes that cause animal African trypanosomiasis (AAT) also express related antigens, we have evaluated two rapid diagnostic tests (RDTs) on cattle in trypanosomiasis endemic and non-endemic regions, to determine whether gHAT serological tests could also be used to screen for AAT.

**Methods:**

Two RDTs, 1G RDT, made with native antigens, and p2G RDT, made with recombinant antigens, were tested on 121 cattle in a trypanosomiasis-free region, and on 312 cattle from a *rhodesiense* HAT and AAT endemic region. A subset of samples from the endemic region were also tested with two immune trypanolysis (TL) tests. The sensitivity of the tests was estimated by evaluating the result of the RDT on samples that were positive by both microscopy and internal transcribed spacer (ITS) PCR, whilst specificity was the result of the RDT on samples that were negative by ITS PCR and microscopy, and others from the non-endemic region.

**Results:**

The specificity of the p2G RDT on cattle from the non-endemic region was 97.5% (95% CI: 93.0–99.2%), compared to only 57.9% (95% CI: 48.9–66.3%) for 1G RDT. The specificities of 1G RDT, p2G RDT and TL on endemic control cattle were 14.6% (95% CI: 9.7–21.5%), 22.6% (95% CI: 16.4–30.3%) and 68.3% (95% CI: 59.6–75.9%), respectively. The sensitivities of the tests on trypanosome positive samples were 85.1% (95% CI: 79.1–89.7%), 89.1% (95% CI: 83.7–93.0%) and 59.3% (95% CI: 51.8–66.4%), respectively. Among the same samples, 51.7% were positive by both TL and the 1G RDT.

**Conclusions:**

These serological tests detect cross-reacting antibodies in cattle. The p2G RDT based on recombinant antigens had a high specificity in a non-endemic region, while the 1G RDT had a lower specificity, suggesting cross-reactivity with other pathogens.

**Electronic supplementary material:**

The online version of this article (10.1186/s13071-017-2487-8) contains supplementary material, which is available to authorized users.

## Background

Human African trypanosomiasis (HAT) and animal African trypanosomiasis (AAT) are endemic in resource-limited regions of at least 36 African countries. Both diseases are caused by protozoan parasites of the genus *Trypanosoma*, which is transmitted mainly by tsetse fly vectors of the genus *Glossina*. The cell surface of bloodstream trypanosomes is covered by a dense monolayer of a single protein coat that consists of millions of copies of a variant surface glycoprotein (VSG) that frequently switches to a new one to enable the parasite to evade the host immune response [1–7]. VSGs are highly immunogenic and are associated with a strong antibody response that is easily detected in circulation [8]. Trypanosomes switch VSGs through differential expression of an archive of hundreds of silent VSG genes and pseudogenes that reconstitute functional genes upon conversion and recombination events. The rate that trypanosomes belonging to one variant antigenic type (VAT) switch expression from one VSG to another is less than 1 in 100 trypanosomes per generation, and as a result, most trypanosomes in a wave of parasitaemia that fail to switch VSGs are destroyed by the host immune system [9–15]. The rate is, however, fast enough for some parasites to switch VSG and become a different VAT, thus evading the host immunological response, to establish the next wave of parasitaemia. Expression of VSGs occurs hierarchically, such that some VATs tend to appear early in infection, others in late disease, and others somewhere in between [11, 14, 16–18]. As a result, in an infected host, there are simultaneously several sub-populations of trypanosomes belonging to different VATs, with estimates that at any point during infection, up to 100 VSGs could be expressed, and as many as 83 have been definitively measured [8, 19, 20]. The variant-specific antibody response tends to persist for long periods during infection, and therefore antibodies to different VATs are found in serum at the same time. One consequence of switching VATs is that there is a constant regeneration of previously expressed variants and their rapid elimination by existing antibodies [14].

Invariant surface glycoproteins (ISGs) are another class of glycoproteins, also attached to the plasma membrane of the trypanosome, but unlike VSGs, they remain unchanged from one cell cycle to another, shielded from host antibodies by the VSG. ISGs also undergo rapid turnover and recycling, with half-lives that are shorter than the generation time of the parasite [21, 22]. The number of copies of individual ISGs is estimated at between 50,000 and 70,000 [23–25], and if this level of expression is extended to the entire ISG family, it is possible that one trypanosome has approximately 200,000 ISGs in total, equivalent to one ISG for every 50 VSG molecules on one trypanosome [26]. In live trypanosomes, most ISGs are shielded from the host immune response by the VSG coat. However, when trypanosomes are killed by a complement-mediated antibody response, the ISGs become exposed, presenting an opportunity for the body to mount an anti-ISG antibody response, making them good candidates for test development [27].

The serological tests used to screen for *gambiense* HAT (gHAT) detect antibodies to VSGs and ISGs [28–30]. These include the card agglutination test for trypanosomiasis (CATT), developed by the Institute of Tropical Medicine (Belgium) [31], first and second generation (1G and p2G) rapid diagnostic tests (RDTs) developed by Alere/Standard Diagnostics (SD; South Korea) [32, 33], and the HAT Sero-K-SeT RDT developed by Coris BioConcept (Belgium) [34]. The CATT test detects antibodies against freeze-dried, fixed trypanosomes expressing VSG LiTat 1.3, while both the 1G SD and Sero-K-SeT RDTs detect antibodies against native VSG LiTat 1.3 and native VSG LiTat 1.5 antigens [35]. The p2G RDT is a prototype developed using recombinant antigens, which detects antibodies to VSG LiTat 1.5 and ISG65 antigens [32]. The immune trypanolysis test (TL), which until now has been used in research settings, is highly specific and has been proposed for use in epidemiological studies, and for surveillance in gHAT elimination programmes [36–38].

This study was carried out to determine whether serological tests for gHAT could also be used in the diagnosis of AAT. We tested cattle in trypanosomiasis endemic and non-endemic regions, to determine whether the tests would exhibit different degrees of cross-reactivity between trypanosome-exposed and non-exposed cattle. We also assessed whether animal-infective trypanosomes express VATs against which an antibody response would be detected by the TL test.

## Methods

### Test samples

**Fig. 1:**
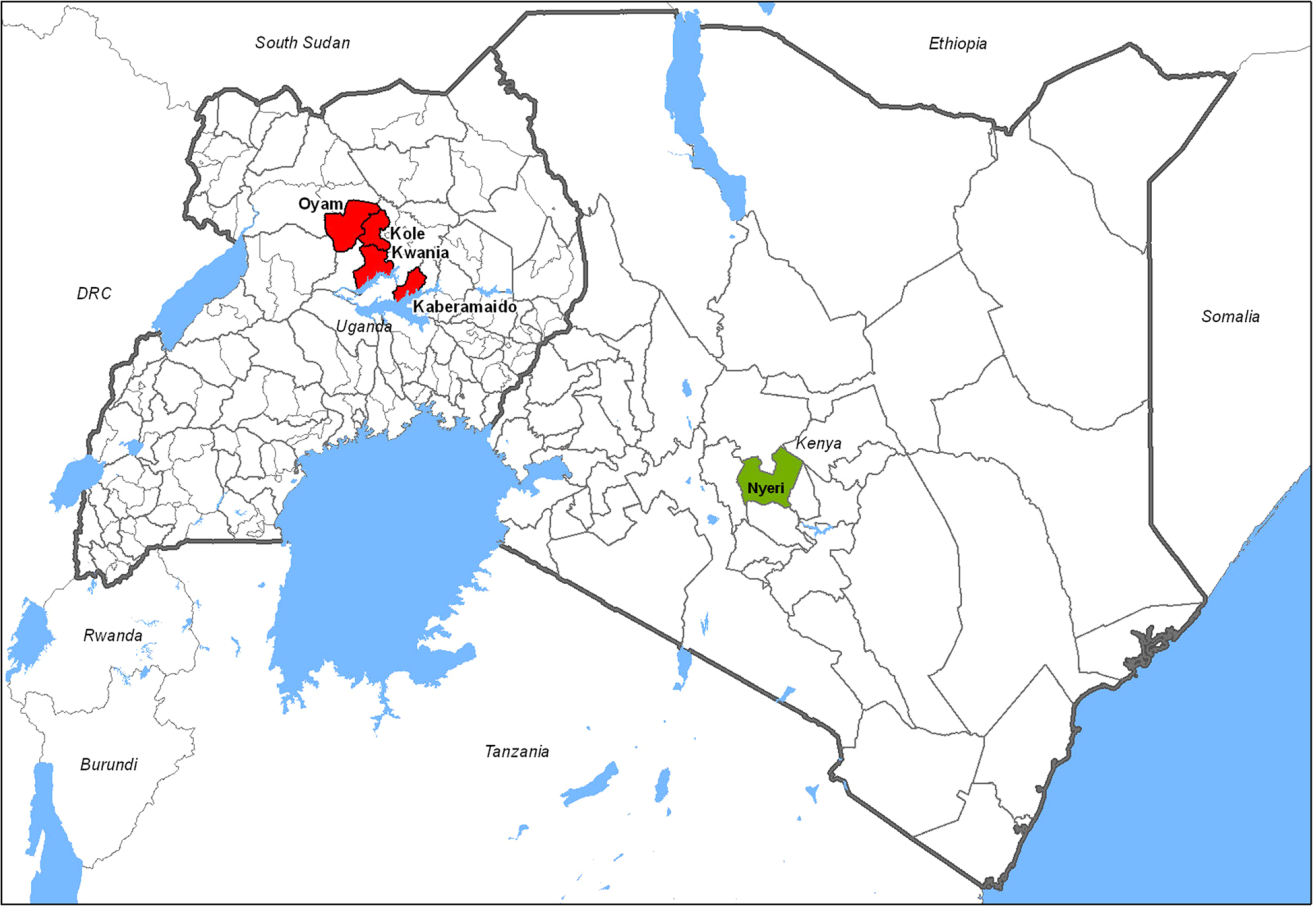
Map of Kenya and Uganda showing the counties of origin of samples analysed (Ugandan samples are shown in red, Kenyan Samples in green). The geographical layers were obtained from CC-BY License compatible sources: GADM (http://www.gadm.org) and the RCMRD geoportal (http://geoportal.rcmrd.org/)

Two categories of samples were used in this study. The first category was blood and sera collected from communally grazed indigenous cattle in a region of mid-northern Uganda that is endemic for *rhodesiense* HAT, and highly endemic for AAT (Fig. 1). The samples were collected in previous surveys of whole herds, comprising animals that were 6 months and older. No data on ages were collected from these animals, but since the animals were grazed communally and whole herds were tested, the majority were more than 12 months old. Venous blood was taken from each animal and immediately examined using the haematocrit centrifugation technique (HCT) to determine the infection status [39]. The blood was stored in liquid nitrogen for subsequent analysis by PCR to determine the parasite species, and to confirm that the animals found negative by HCT were indeed negative (endemic controls). The second category of samples comprised fresh blood obtained by an ear prick from 121 cattle owned by small-scale farmers in a region of central Kenya that is free from tsetse and trypanosomiasis (non-endemic controls). The animals were mainly exotic and cross-bred, aged from 1 week to 10 years, and kept at different levels of husbandry, some in well managed zero-grazing systems in which calves were separated from cows, and others in open paddocks. Of these, 39 (32.0%) were less than 1 year old, 41 (33.6%) between one and 4 years, and 42 (34.4%) were 4 years or older.

### Test kits

**Fig. 2:**
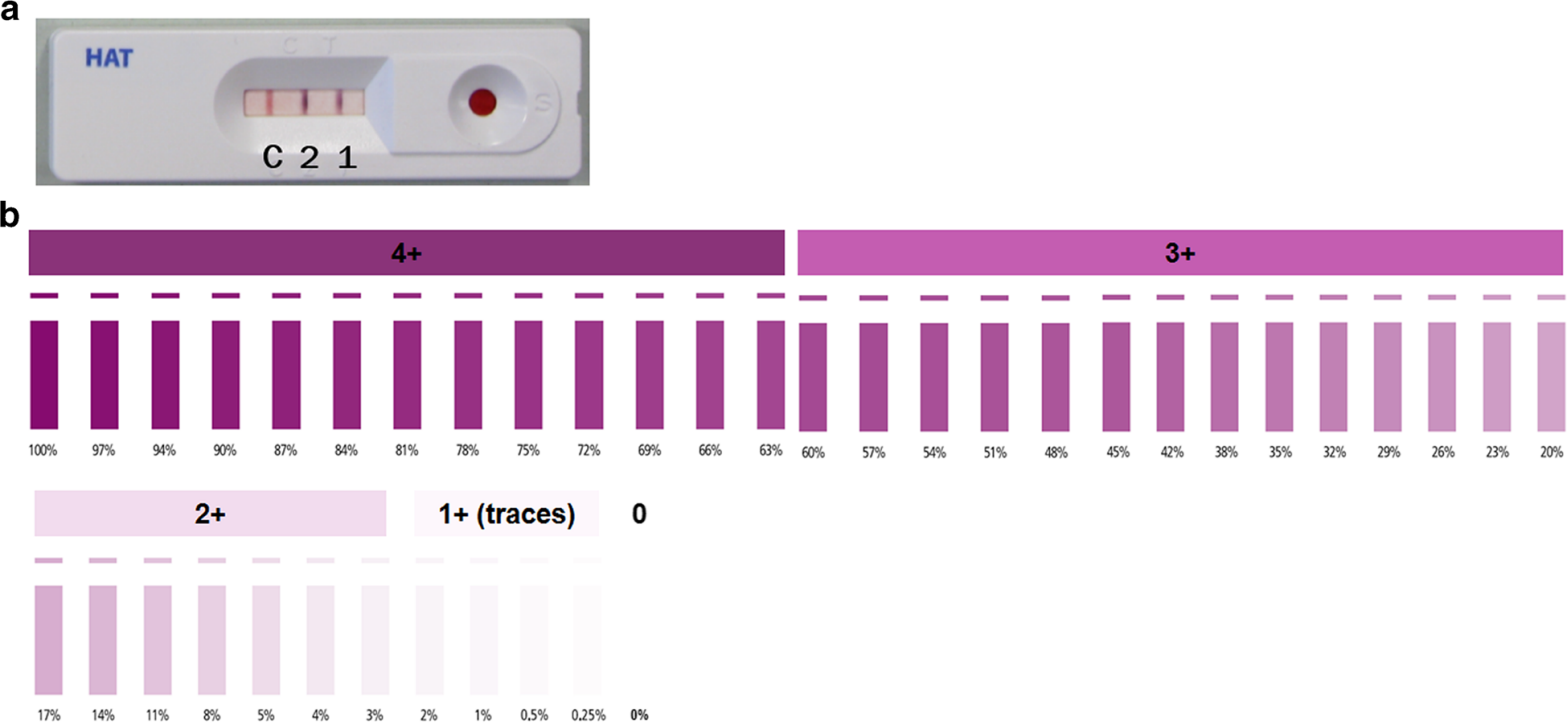
A 1G RDT for gHAT (**a**) and colour chart (**b**) for estimating the intensity of positive results. The RDT shows positive results on both antigen bands (1 and 2) after testing fresh blood. C, control band

Two gHAT RDTs developed by SD were used in the study. The 1G RDT, named SD BIOLINE HAT, was developed using native VSGs (Nat-LiTat 1.3 and Nat-LiTat 1.5) obtained from the Institute of Tropical Medicine (ITM) in Antwerp, Belgium, and detects anti-VSG LiTat 1.3 (band 1) and anti-VSG LiTat 1.5 (band 2) antibodies [32, 33, 35]. The p2G RDT is a prototype developed using the recombinant antigens rISG65 (band 1) and rLiTat 1.5 (band 2) and detected the corresponding antibodies to ISG65 and VSG LiTat 1.5 [32]. The formats of both tests allow reading of results of each antigen individually (see the example of 1G RDT in Fig. 2). The intensity of positive test bands is estimated using a colour chart provided by the manufacturer. When the RDTs are used to screen patients, however, a test is considered positive if the result of any of the two antigens is positive.

### Test procedures

#### Detection of parasite DNA

Whole blood samples collected in Uganda were thawed and DNA extracted using a DNeasy Blood and Tissue Kit (Qiagen, Hilden, Germany), then tested by PCR targeting the internal transcribed spacer (ITS1) as described by Njiru et al. [40]. For this study, cases were defined as cattle that were positive by both microscopy and ITS PCR; endemic controls were animals from the same herds that were negative by both microscopy and PCR. Samples whose parasitological and ITS PCR results did not agree were excluded in the subsequent analysis. A subset of samples whose ITS PCR indicated the presence of the *T. brucei* group (*Trypanozoon*) were tested further using the serum resistance associated PCR (SRA-PCR) and the *T. gambiense*-specific glycoprotein PCR (TgsGP-PCR) [41] to determine the presence of *T. b. rhodesiense* or *T. b. gambiense*, respectively. These tests were not performed on the non-endemic control samples from Kenya because the study region is free from tsetse and trypanosomiasis.

#### Rapid diagnostic tests

The rapid diagnostic tests were performed according to the manufacturers’ instructions. Briefly, in Kenya, 20 μl of fresh blood samples from 121 cattle were obtained from an ear-prick and immediately put in the sample wells of both RDTs in parallel. Four drops of buffer were added, and the results were read after 15 to 20 min. In Uganda, the serum was thawed and 10 μl used to perform the test. The technician who performed the tests on samples from Uganda was blinded to their identity. In both countries, the result for each antigen was recorded and when positive, the intensity of the bands was estimated using a colour scale, from +1 (lowest) to +4 (highest) (Fig. 2b). The results were also recorded digitally by taking a photograph.

#### Immune trypanolysis tests

The immune trypanolysis tests (TL) were performed only on samples from the endemic region, as they only detect antibodies to trypanosomes. A total of 188 frozen blood and 163 serum samples were shipped to the Institute of Tropical Medicine (ITM) in Antwerp, Belgium. The samples were tested for antibodies against VSG LiTat 1.3 and VSG LiTat 1.5 using two TL tests as described by Van Meirvenne et al. [9]. Briefly, 25 μl of the undiluted test sample was pipetted into separate wells of microplates. 25 μl of complement-rich guinea pig serum (GPS) was added and plates pre-incubated at room temperature for 30 min. Next, 50 μl of a 10^7^/ml suspension of trypanosomes in GPS were added and plates incubated at room temperature for another 90 min. A drop of the reaction mixture was then examined under a phase contrast microscope (250×). The highest antiserum dilution, defined as the final dilution in the reaction mixture still causing 50% lysis of trypanosomes was arbitrarily considered as the end-point titre, and samples with a higher figure were recorded as positive. A sample was recorded as positive if either of the TL tests was positive. Only samples with sufficient volumes for at least one of the TL tests were included in this assay. The testing laboratory was blinded to the origin of the samples.

#### Case/control definitions and statistical methods

For this study, ‘cases’ were defined as cattle that were positive by both microscopy and ITS PCR; ‘endemic controls’ were animals from the same herds that were negative by both microscopy and PCR. Any samples from the endemic region for which the results of microscopy and ITS PCR were discordant were excluded. ‘Non-endemic controls’ were also included in the analyses.

All statistical analyses were performed in the R statistical environment [42]. 95% confidence intervals (CI) were calculated using the Wilson interval. Statistical differences between the percentage of tests that were positive and those negative on unpaired tests were evaluated using Fisher’s exact test and using McNemar’s chi-square test on paired 2 × 2 table data. The intensity of the RDT bands was analysed by converting the categorical scale to a numeric scale with 0 = negative, 1 = 1+ to 4 = 4 + based on the manufacturers’ chart (Fig. 2b).

## Results

### Microscopy and PCR on endemic samples

The samples were from two batches. In the first batch, 194 samples were pre-selected from a larger set of samples and comprised 99 that were positive by both ITS1 PCR for *T. brucei*, as well as microscopy and so, were included as cases, while 95 were negative by both tests and so were therefore included as endemic controls.

The second batch of 159 samples included samples that were positive with other animal infective trypanosome species (*T. congolense* and *T. vivax*) that co-exist with *T. brucei*. For this, we tested the 159 samples with no pre-selection, including 76 that met the case definition criteria; 42 controls, and 41 that gave discordant results and were therefore excluded. Of the 76 cases in this batch, 47 (61.8%) were positive for only one species (*T. brucei*, *T. congolense* or *T. vivax*), 16 (21.1%) were positive for any two, while 13 cattle (17.1%) were positive for all the three species. Overall in the second batch, 31 animals (40.8%) were infected with *T. brucei*, either as a single infection or as a co-infection with other species (Additional file 1: Table S1). In total, there were 175 cases and 137 controls from the endemic region.

### Performance of serological tests

#### RDT positivity

When 121 samples from the non-endemic region were tested with the 1G RDT, 51 (42.1%; 95% CI: 33.7–51.1%) were positive on at least one band (Nat-LiTat1.3 and/or Nat-LiTat 1.5). This was significantly lower (Fisher’s exact test, *P* < 0.001) than the 266 out of 312 (85.3%; 95% CI: 80.9–88.8%) samples from the endemic region that were positive by the 1G RDT. Furthermore, the band intensities (a qualitative indication of the concentration of detectable antibodies) of the positive samples from the non-endemic region were fainter (in the range 1.07–1.45), compared to those from the endemic sera (range 2.07–2.44) (Table 1).

**Table 1:** Percentage positivity and intensity of positive bands of the 1G HAT RDT on cattle samples from AAT endemic and non-endemic regions

	Cases (*n* = 175)		Endemic controls (*n* = 137)		Non-endemic controls (*n* = 121)	
Device	% positive (95% CI)	Mean band intensity ± SD (+ve’s)	% positive (95% CI)	Mean band intensity ± SD (+ve’s)	% positive (95% CI)	Mean band intensity ± SD (+ve’s)
1G RDT (either band +ve)	85.1 (79.1–89.7) (*n* = 149)	2.15 ± 1.11	85.4 (78.5–90.3) (*n* = 117)	2.07 ± 1.09	42.1 (33.7–51.1) (*n* = 51)	1.07 ± 0.46
Band 1 (Nat-LiTat 1.3)	81.1 (74.7–86.2) (*n* = 142)	2.44 ± 1.11	76.6 (68.9–82.9) (*n* = 105)	2.35 ± 1.13	38.8 (30.6–47.7) (*n* = 47)	1.45 ± 0.50
Band 2 (Nat-LiTat 1.5)	72.6 (65.5–78.6) (*n* = 127)	2.31 ± 1.15	71.5 (63.5–78.4) (*n* = 98)	2.42 ± 1.14	24.0 (17.2–32.3) (*n* = 29)	1.41 ± 0.50

*Abbreviation*: *SD* standard deviation

There was a significant difference between the results of bands 1 and 2 of the 1G RDT in the non-endemic samples (Tables 2 and 3; McNemar’s *χ* ^2^_1_ = 11.1, *P* < 0.001) with greater numbers positive on band 1 (Nat-LiTat 1.3), and only four positives on band two alone. Similar patterns were seen in the samples from the endemic region.

**Table 2:** Agreement between bands 1 and 2 in the 1G RDT on samples from Kenya and Uganda

1G RDT			Band 2 LiTat 1.5	
			-ve	+ve
Band 1 LiTat 1.3	Non-endemic samples (*n* = 121)	-ve	70	4
		+ve	22	25
	Endemic samples (*n* = 312)	-ve	46	19
		+ve	41	206

Only 3 out of 121 cattle (2.5%; 95% CI: 0.8–7.0%) from the non-endemic region were positive with the p2G RDT (Table 4). Two of the three animals, aged 15 months and 4 years, were positive in band 1 (rlSG65) only. The third, aged 10 years, was positive on band 2 (rLiTat 1.5). Significantly fewer were positive on band 2 (rLiTat 1.5) in the p2G RDT (0.8%; 95% CI: 0–4.5%) than band 2 (Nat-LiTat 1.5) in the 1G RDT (24.0%; 95% CI: 17.2–32.3%) yet both bands detect antibodies to the same antigen (McNemar’s *χ* ^2^_1_ = 5.31, *P* = 0.021). These results were significantly different from the results on sera from the endemic region (Fisher’s exact test, *P* < 0.001), in which 89.1% of cases and 77.4% of controls were positive by the p2G RDT, mainly due to band 1 (rISG65) (Table 4). A significantly larger proportion of the sera were positive in band one compared to band 2 of the p2G RDT in samples from the endemic region (McNemar’s *χ* ^2^_1_ = 25.8, *P* < 0.001).

**Table 3:** Agreement between bands 1 and 2 in the p2G RDT on samples from Kenya and Uganda

p2G RDT			Band 2 rLiTat 1.5	
			-ve	+ve
Band 1 rlSG65	Non-endemic samples (*n* = 121)	-ve	118	1
		+ve	2	0
	Endemic samples (*n* = 312)	-ve	50	15
		+ve	60	187

**Table 4:** Percentage positivity of the p2G HAT RDT on cattle sera from the endemic and non-endemic regions (S2)

	Cases (*n* = 175)		Endemic controls (*n* = 137)		Non-endemic controls (*n* = 121)	
	% positive (95% CI)	Mean band intensity ± SD (+ve’s)	% positive (95% CI)	Mean band intensity ± SD (+ve’s)	% positive (95% CI)	Mean band intensity (+ve’s)
p2G RDT	89.1 (83.7–92.9) (*n* = 156)	2.16 ± 0.94	77.4 (69.7–83.6) (*n* = 106)	1.69 ± 0.86	2.5 (0.8–7.0) (*n* = 3)	0.67
Band 1 (ISG65)	86.3 (80.4–90.6) (*n* = 151)	2.83 ± 1.08	70.1 (61.9–77.1) (*n* = 96)	2.36 ± 1.16	1.7 (0.5–5.8) (*n* = 2)	1
Band 2 (rLiTat 1.5)	66.9 (59.6–73.4) (*n* = 117)	2.10 ± 1.05	62.0 (53.7–69.7) (*n* = 85)	1.54 ± 0.80	0.8 (0–4.5) (*n* = 1)	2

*Abbreviation*: *SD* standard deviation

Standard deviation is not shown for the endemic control region owing to the small number of positives

The results of testing sera from cattle in an endemic region with the 1G and p2G RDTs are shown in Table 5. There was no significant difference in the percentage positivity between cases (85.1%) and endemic controls (85.4%) (Fisher’s exact test, *P* = 1). Among the cases, 168 (96%) were positive by one or both RDTs, while among the endemic controls, 121 (89.1%) were positive with one or both RDTs, altogether, 290 (92.9%) animals in the endemic area were positive with one or both RDTs.

**Table 5:** Some endemic sera that were positive by individual antigens and RDTs

RDT	Infection status	No. of samples	RDT	By RDT band		
			Either band	Band 1 only	Band 2 only	Bands 1 & 2
1G	Cases	175	149 (85.1%)	22 (12.6%) (Nat-LiTat 1.3)	7 (4.0%) (Nat-LiTat 1.5)	120 (68.6%)
	Endemic controls	137	117 (85.4%)	19 (13.9%) (Nat-LiTat 1.3)	12 (8.8%) (Nat-LiTat 1.5)	86 (62.8%)
	Total	312	266 (85.3%)	41(13.1%)	19 (6.1%)	206 (66.0%)
p2G	Cases	175	156 (89.1%)	39 (22.3%) (rISG65)	5 (2.9%) (rLiTat 1.5)	112 (64.0%)
	Endemic controls	137	106 (77.4%)	21 (15.3%) (rISG65)	10 (7.3%) (rLiTat 1.5)	75 (54.7%)
	Total	312	262 (84.0%)	60 (19.2%)	15 (4.8%)	187 (59.9%)

When the results of native and recombinant VSG LiTat 1.5 were compared, Nat-LiTat 1.5 (band 2 of the 1G RDT) was positive in a larger number of cases (127, comprising 7 on band 2 only and 120 on bands 1 and 2, i.e. 72.6%) than rLiTat 1.5 (band 2 on the p2G RDT) (117, comprising 5 on band 2 only and 112 on bands 1 and 2, i.e. 66.9%). The difference was however not significant (McNemar’s *χ* ^2^_1_ = 1.45, *P* = 0.229). Similarly, among endemic controls, 98 (comprising 12 on band 2 only and 86 on bands 1 and 2, i.e. 71.5%) sera were positive for Nat-LiTat 1.5 (band 2 on the 1G RDT) compared to 85 (comprising 10 on band 2 only and 75 on bands 1 and 2, i.e. 62.0%) for rLiTat 1.5 (band 2 of the p2G RDT), a difference that was significant (McNemar’s *χ* ^2^_1_ = 4.11, *P* = 0.045) (Table 5).

A comparison of the results of individual antigens on both RDTs with PCR and microscopy are shown in Table 6. The results for both RDTs agreed in 56% (*n* = 7+ 4 + 87 = 98) of cases and 62.0% (*n* = 15 + 5 + 4 + 61 = 85) of endemic controls. 148 (87 + 61) (47.4%) cattle were positive with all four antigens; 87 (49.7%) of these were cases and 61 (44.5%) endemic controls.

**Table 6:** Comparison of results of antigens on the 1G and p2G RDTs on samples from the endemic area

		p2G RDT							
		Cases (*n* = 175)				Endemic controls (*n* = 137)			
		−/−	+/−	−/+	+/+	−/−	+/−	−/+	+/+
1G RDT	−/−	7	10	0	9	15	3	0	2
	+/−	4	4	0	14	5	5	3	6
	−/+	0	5	0	2	0	2	4	6
	+/+	8	20	5	87	11	11	3	61

*Abbreviations*: −/−, negative both bands; +/−, positive band 1 only; −/+, positive band 2 only; +/+, positive both bands

The number and intensity scores of positive bands in the p2G RDT were higher on band 1 (rISG65) than band 2 (rLiTat 1.5), particularly among cattle that were trypanosome positive (Wilcoxon rank sum test, *P* < 0.001). This difference in intensity scores was not observed with the 1G RDT (*P* = 0.618) (Tables 1 and 4).

Five of the *T. brucei (Trypanozoon)* positive samples from Uganda (*n* = 130) that were tested with SRA-PCR were positive, indicating the presence of *T. b. rhodesiense,* while the other 125 were *T. b. brucei*. These five were also positive by both bands of the p2G RDT. Four of these samples were positive by both bands of the 1G RDT and one by band one only. None of these samples was positive with TgsGP-PCR (*T. b. gambiense*).

### Effect of age on the positivity of RDTs

**Fig. 3:**
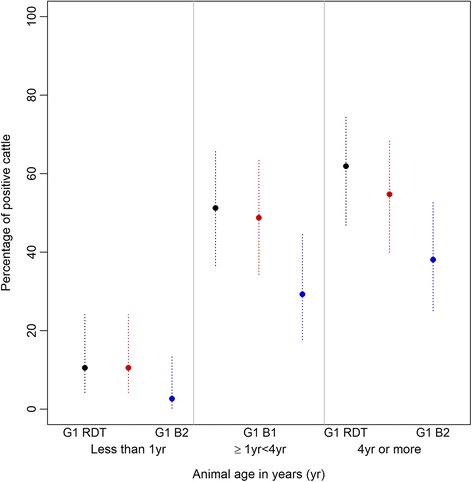
Percentage and 95% confidence interval of samples found positive with 1G RDT in cattle in a non-endemic region of Kenya. The results are shown by age group, overall RDT result and individual bands

The number of cattle in the non-endemic region that were positive with the 1G RDT increased with age (Fig. 3). There was a significant difference on both antigens separately, and on the overall RDT result (Fisher’s exact *P*-value < 0.001) between animals aged less than 12 months and those that were 12 months or older. In the older age groups (12 months and older), the percentage positivity was similar to that seen in cattle in the endemic region, but the band intensity among positive animals in this age group remained low (1.41 and 1.40 in bands 1 and 2). The effect of age on the results of the p2G RDT could not be assessed because only three animals were positive with this RDT.

### Immune trypanolysis tests on sera from the endemic region

The immune trypanolysis (TL) tests were performed on 295 sera from Uganda, including 172 that were cases, and 123 endemic controls (Table 7). Of the 295, 141 (47.8%) were positive by TL (comprising 102 cases and 39 endemic controls). Among the sera from cases, TL Nat-LiTat 1.5 was positive in 52.3%, while 82.9% of endemic controls tested negative, the difference in numbers testing positive (52.3% of cases vs 100–82.9% = 17.1% endemic control sera) was statistically significant (Fisher’s exact *P*-value <0.001). However, TL Nat-LiTat 1.3 was positive in only 23.3% of the case sera, but the specificity among the endemic control sera was 79.7%, the difference in numbers testing positive (23.3% of cases vs 100–79.7% = 20.3% endemic control sera) was not significant (Fisher’s exact *P*-value = 0.572). The high sensitivity among trypanosome positive sera was mainly driven by VSG LiTat 1.5 (Tables 7, 8) (McNemar’s *χ* ^2^_1_ = 32.4, *P* < 0.001) with 23.3% positive by VSG LiTat 1.3 and 52.3% by VSG LiTat 1.5, but there was no difference among the negative sera (McNemar’s *χ* ^2^_1_ = 0.281, *P* = 0.60), corresponding specificities were 79.7 and 82.9%.

**Table 7:** Results of the TL tests on cattle sera from the endemic region

	Cases			Endemic controls		
TL result	TL^a^	TL Nat-LiTat 1.3	TL Nat-LiTat 1.5	TL^a^	TL Nat-LiTat 1.3	TL Nat-LiTat 1.5
Tested	172	172	172	123	123	123
Negative	70	132	82	84	98	102
Positive	102	40	90	39	25	21
Sensitivity (%) (95% CI)	59.3 (51.8–66.4)	23.3 (17.6–30.1)	52.3 (44.9–59.7)	–	–	–
Specificity (%) (95% CI)	–	–	–	68.3 (59.6–75.9)	79.7 (71.7–85.8)	82.9 (75.3–88.6)

^a^Results of both TL tests combined

**Table 8:** Cross-tabulation of TL results by antigen

		TL Nat-LiTat 1.5		
		Negative	Positive	Total
Cases				
TL Nat-LiTat 1.3	Negative	70	62	132
	Positive	12	28	40
	Total	82	90	172
Endemic controls				
TL Nat-LiTat 1.3	Negative	84	14	98
	Positive	18	7	25
	Total	102	21	123

As both TL tests detect antibodies against the antigens that are included in the 1G RDT, results of TL were compared to those obtained using the 1G RDT. Among cases, 51.7% were also positive by both TL and the 1G RDT, with the highest agreement being due to the VSG Nat-LiTat 1.5 (Table 9). Among endemic controls, a smaller number (30.9%) were positive by both 1G RDT and by TL.

**Table 9:** Results of TL tests and 1G RDT, and the level of agreement between tests

Test^a^	Positive TL (%)	Positive RDT (%)	Positive agreement (%)	Negative agreement (%)
Cases (*n* = 172)				
Test	102 (59.3)	148 (86.0)	89 (51.7)	11 (6.4)
Nat-LiTat 1.3	40 (23.3)	141 (82.0)	36 (20.9)	27 (15.7)
Nat-LiTat 1.5	90 (52.3)	127 (73.8)	69 (40.1)	24 (14.0)
Endemic controls (*n* = 123)				
Test	39 (31.7)	105 (85.3)	38 (30.9)	17 (13.8)
Nat-LiTat 1.3	25 (20.3)	94 (76.4)	22 (17.9)	26 (21.1)
Nat-LiTat 1.5	21 (17.1)	89 (72.4)	20 (16.2)	33 (26.8)

^a^“Test” refers to the test being positive, in TL and the RDT, the test is interpreted as positive if either band is positive. Nat-LiTat 1.3 and 1.5 refer to the results of each antigen in TL and the RDT. Positive and negative agreement are the numbers where the test results agreed

## Discussion

This study has demonstrated a variability of the specificity of RDTs for gHAT when tested on cattle, depending on the antigens used, and on the presence or absence of AAT in a region. The specificity of the p2G RDT, which is made using recombinant antigens, on cattle in a non-endemic region was very good. In contrast, the specificity of the 1G RDT, made using native antigens, decreased with the age of the animals. However, when the three serological tests used in this study were performed on samples from cattle in an AAT endemic region, they all had a low specificity. Antibodies to ISG65 were detected in the largest number of cattle in the endemic region. This could be because, unlike VSGs, ISG65 is constantly expressed by all trypanosome populations in the host, unlike VSGs, which switch with each wave of parasitaemia; the ISG family was previously investigated as *T. congolense* diagnostics with promising results [43]. In live trypanosomes, ISGs are largely protected from the host immune response by the VSG coat. However, as parasites are continuously killed by the host anti-VSG antibody response, this process exposes ISGs, eliciting a persistent antibody response, which could explain the larger number of positive cattle.

The results of this study also suggest that all the antigens tested are likely to have cross-reacting epitopes from other trypanosome species, but perhaps not predominantly. This would not be surprising, considering that recently, a high similarity between different trypanosome species has been demonstrated at the genomic level [44, 45]. In other studies, the CATT test, which has the VSG LiTat 1.3 antigen, was also positive in animals infected with either *T. congolense, T. b. brucei* or *T. evansi* [46–48]. In another study, a large number of trypanosome antigens, including VSG LiTat 1.3 and VSG LiTat 1.5, reacted to sera from both *T. b. gambiense* and *T. b. rhodesiense* patients [49]. Another candidate, rISG65-2 had a sensitivity of 92% and a specificity of 85% for *T. b. rhodesiense* [50, 51], indicating that, while the antigens in these studies had good sensitivity for both *T. b. gambiense* and *T. b. rhodesiense*, their sensitivity was not restricted to the two sub-species of trypanosomes.

The specificity of the recombinant antigens on non-endemic cattle was very good, while that of native antigens was low. As a result, the p2G RDT had a very good specificity when compared to the 1G RDT. This difference may be attributed to the manner in which the antigens used to develop the tests are produced. The VSGs used in the 1G RDT are produced from pathogenic *T. b. gambiense* in a process that involves infecting rodents and harvesting the parasites during the first peak of parasitaemia. The parasites are purified by gel chromatography, and the VSG is separated through a process of freeze-thawing and fractionation [52]. While the final sample would comprise mainly VSGs of the particular VAT, proteins that could cross-react with antigens expressed by related organisms would likely be present. The possibility of such cross-reactivity would explain the lower specificity of the 1G RDT on samples from the non-endemic region. In that husbandry system, cattle were under different levels of exposure to infections such as anaplasmosis, theileriosis and babesiosis; and besides, the presence of the tabanid-transmitted *T. theileri* cannot be ruled out. This hypothesis is supported by our observation that the likelihood of an animal in the non-endemic region being positive with the 1G RDT increased with age. In the non-endemic region, young calves are usually kept in sheds where the risk of being bitten by ticks that transmit such diseases is low. As the calves grow older, they are kept in zero-grazing systems or in open paddocks in close contact with older animals, where the risk of tick infestation and transmission of disease is comparatively higher. Indeed, previous reports have suggested a possible cross-reactivity of the CATT reagents with antibodies against other infections such as malaria [53, 54]. There could, however, be other reasons for the low specificity of the 1G RDT in these animals, such as the likely presence of anti-VSG native antibodies in animals that had never been exposed to trypanosomes, a phenomenon that has been observed in mice, bovines and humans [53]. Noteworthy is that although LiTat1.3 is considered specific to *T. b. gambiense* it is identical to many other *Trypanozoon* VSGs. A BLAST search with the respective protein sequence (accession no. AHW98113.1) indicates considerable identity with several *T. brucei* VSGs, peaking at 79–81% with four different VSGs (accession nos. APD74596.1, APD75145.1, AGH60298.1, APD73903.1, AGH59165.1). The search also picks out putative VSGs from *T. equiperdum* albeit at lower identity (40–45%). Thus cross-reactivity should be expected even with other trypanosomes expressing different VSGs. Besides, all VSGs share the so-called cross-reactive determinant (CRD) epitope on the GPI anchor that can also contribute to cross-reactivity.

Unlike the 1G RDT, the p2G RDT is made using highly purified recombinant antigens, which could explain the high specificity observed in cattle from the non-endemic region. Indeed when the results of the native VSG LiTat 1.5 antigens are compared with those of the recombinant variant, the native antigen was positive in more samples than the recombinant protein in both the endemic and non-endemic regions, regardless of the infection status, although the difference was not significant. The additional positive samples with native antigens in the endemic region could be due to cross-reactivity with antibodies against other pathogens, otherwise the native and recombinant forms of the same antigen detect the same antibodies.

Among the trypanosome positive samples, more than half were also positive by TL, a clear indication that TL positive results are associated with trypanosome infection. A proportion of endemic controls were also positive by TL. As with RDTs, it is likely that some of these controls were true infections that were missed by microscopy and PCR, attributed to the higher sensitivity of tests that detect host antibodies, which would be abundant in blood.

The number of trypanosome positive and negative samples from the endemic region that were positive with one or both RDTs was not significantly different. This could be confusing, considering that animals were identified as negative when the results of microscopy and PCR were negative. However, RDTs detect antibody responses to exposure to parasites, and not necessarily active infection. This, coupled with a lower sensitivity of parasitological and molecular tests when compared to detection of antibodies, means that some of the animals that were RDT positive but trypanosome negative could have been missed infections. That some animals had an antibody response to each of the test antigens may be related to the duration of infection. Waves of parasitaemia are associated with different VATs, and the longer an animal remains infected, the more VATs appear in the blood, each eliciting an antibody response [30, 33, 36]. Sometimes farmers also treat their animals with trypanocides, and in such cases, recently treated animals would be positive for antibodies but trypanosome negative.

The p2G RDT detected 89.1% of the sera from the endemic region that were trypanosome positive, which was significantly higher than those positive among the endemic controls. This is a striking correlation, considering that at present, no RDT for diagnosis of AAT is commercially available. The possibility that this test could be used for passive screening of cattle that have clinical signs in AAT endemic regions needs further investigation. The test would not be used for herd screening since not all animals with an antibody response would develop the disease. In the context of elimination of both gHAT and AAT however, such a test would be useful for confirming total clearance of trypanosomes in a region, and in detecting likely re-emergence when used on sentinel herds.

The sensitivity of the 1G RDT in trypanosome positive samples was higher than TL, which uses live parasites expressing the same VSGs as those used to develop the RDT. Indeed when the results of each of the TL tests were considered separately, the sensitivity decreased further. This would be expected, since TL detects antibodies that are specific to the VATs in the test [11], in this case only anti-VSG LiTat 1.3 and anti-VSG LiTat 1.5 antibodies. A similar difference between TL tests and other screening tests has been reported in other studies [47]. The difference in sensitivity between the 1G RDT and TL could be because the VSGs used to develop the 1G RDT are detached from trypanosomes, exposing epitopes that in the live parasites used in the TL test, would not be available to detect antibodies against them [52]. In the animal, however, such epitopes would have been exposed when trypanosomes are killed by the host immune system, eliciting an antibody response that would be detected by the RDT but not by the TL test.

The sensitivity of TL was largely driven by TL Nat-LiTat 1.5. The comparatively low sensitivity of TL Nat-LiTat 1.3 could be an indication that this antigen is expressed at a lower frequency by trypanosomes that cause AAT than the other antigens used in this study. *T. b. gambiense* parasites expressing LiTat 1.3 have in the past been among the most suitable for developing a gHAT screening test as opposed to those expressing LiTat 1.5 or LiTat 1.6 [31, 37, 52, 55]. The TL tests were the least sensitive among the serological tests used in the present study, which is understandable, because the tests are VAT-specific, only detecting antibodies to the specific parasite VATs. A similar low sensitivity of TL has been reported in other studies [17].

The low specificity of the 1G RDT observed in a non-endemic region could have implications on the interpretation of positive screening test results in animals living in gHAT endemic regions. In previous studies in gHAT endemic regions, animals found positive with the CATT test were considered to be infected with *T. b. gambiense,* and therefore potential reservoirs of the disease [48]. The results of this study, however, show that besides the possible existence of *T. b. gambiense* in the animal reservoir, cross-reactivity with other infections or with host proteins could influence the performance of the test. Conversely, the rLiTat 1.5 antigen on the p2G RDT was positive in only one animal (99.2% specificity) in the non-endemic region, indicating that in a region that is endemic for trypanosomiasis, a positive result with this antigen could most likely be due to the presence of anti-trypanosome antibodies. Other molecular methods such as sub-species-specific PCR would, however, be required to confirm the presence of human-infective trypanosomes in such samples.

Five of the *T. brucei* positive samples from the endemic region were also positive with SRA-PCR, indicating the presence of *T. b. rhodesiense,* further evidence that livestock is an important reservoir of *T. b. rhodesiense* HAT [39, 56]. None of the samples was positive with TgsGP-PCR, indicating that *T. b. gambiense* was not circulating in the region, and further indirect evidence that the results obtained with the RDTs were due to the presence of cross-reacting antibodies to parasites other than *T. b. gambiense*.

This study has demonstrated that serological tests for gHAT when used on cattle, cross-react with antibodies to other pathogens. These results, if reproduced in humans, could have important implications on the selection of samples suitable for development of tests for gHAT, interpretation of screening test results, control of the disease, and surveillance to sustain elimination. Plasma and serum samples that are used during development of RDTs for gHAT are taken from people living in regions that are endemic for both gHAT and AAT. The distinction between the samples has been that gHAT cases are those that are confirmed by microscopy, in most cases following a positive screening test, while controls are individuals found negative by screening tests that are in use at that time. Current screening tests detect antibodies to only one or two antigens, while there are likely to be many more anti-trypanosome antibodies in the samples, depending on the number of VATs that had been expressed in different waves of parasitaemia, and to exposure to various trypanosome species. Such antibodies could be detected by prototype tests during development, with the risk that the prototypes would be discarded on account of low specificity. Indeed in a recent study in which newly developed RDTs were tested using stored samples, their specificity was found to be low, while the sensitivity was very good [35]. The control samples in that study had been classified as controls because they were negative with CATT, which mainly detects antibodies to VSG LiTat 1.3. To minimize such challenges, we recommend that control samples for development of serological tests be obtained from regions that are geographically similar to gHAT endemic ones, but free from tsetse flies, and both HAT and AAT.

Clinical testing of new tests for gHAT is carried out at multiple sites, to correct for the potential geographic variability of trypanosome strains. Due to the continued decline in the number of gHAT cases reported annually, data from multiple sites is pooled to meet the required number of study participants. The specificity of such tests has often varied from region to region [33, 35, 55]. The results of this study show that it is possible to have antibody responses to trypanosomes that do not cause gHAT, and to non-target organisms, which should be taken into account when designing clinical trials on new tests.

This study had some limitations. The serological tests are designed for use on human samples. Additional studies are needed, to determine whether their performance is affected by factors that could be present in cattle serum. The communally grazed cattle in the AAT endemic region are occasionally treated with trypanocides, and due to the persistence of antibodies, such animals would have been positive with the serological test but trypanosome negative, creating a bias on the number of endemic controls that were positive by serology. The TL tests were included to determine whether they can detect VAT-specific antibodies to trypanosomes that are not infective to humans, and because they are highly specific to trypanosomes, they were not included in the samples from the non-endemic region. This omission could have biased the TL results and was a missed opportunity to confirm the specificity of the tests.

## Conclusions

While the specificity of an RDT for gHAT made using recombinant antigens was very good when used on cattle in a trypanosomiasis-free region, this decreased dramatically in an AAT endemic region, limiting its potential as a diagnostic test. The specificity of an RDT made using native parasite antigens decreases with the age of the cattle, probably due to cross-reacting antibodies arising from exposure to other pathogens. Trypanosomes that are not infective to humans are capable of switching to VATs that are detectable by the current serological tests for gHAT, rendering the tests of less utility for identification of animal reservoirs for gHAT.

## Additional file

Additional file 1: Table S1. Results of ITS1-PCR on the 76 PCR and parasitology positive samples from the second set of sera. (DOCX 15 kb)

## Abbreviations

1G: First generation
AAT: Animal African trypanosomiasis
CATT: Card agglutination test for trypanosomiasis
CI: Confidence interval
ELISA: Enzyme-linked immunosorbent assay
gHAT: *gambiense* human African trypanosomiasis
GPS: Guinea pig serum
HAT: Human African trypanosomiasis
HCT: Haematocrit centrifugation technique
ISG65: Invariant surface glycoprotein 65
ITM: Institute of Tropical Medicine
ITS: Internal transcribed spacer
Nat: Native
p2G: Prototype second generation
RDT: Rapid diagnostic test
rHAT: *rhodesiense* human African trypanosomiasis
SD: Alere/standard diagnostics
SRA: Serum resistance associated
TgsGP: *T. gambiense-*specific glycoprotein
TL: Immune trypanolysis tests
VAT: Variant antigenic type
VSG: Variant surface glycoprotein
