# Supplementary material for: Serological tests for gambiense human African trypanosomiasis detect antibodies in cattle
Source: Parasit Vectors. 2017 Nov 3;10:546. doi: 10.1186/s13071-017-2487-8 (PMC5670715; doi:10.1186/s13071-017-2487-8)
Supplement: Additional file 1: Table S1. — Results of ITS1-PCR on the 76 PCR and parasitology positive samples from the second set of sera. (DOCX 15 kb) [file 13071_2017_2487_MOESM1_ESM.docx]

**Additional file 1: Table S1**. Results of ITS1-PCR on the 76 PCR and parasitology positive samples from the second set of sera.

| **Trypanosome species** | **Number positive (%)** |
| --- | --- |
| *T. brucei* only | 6 (7.9) |
| *T. congo* only | 16 (21.1) |
| *T. vivax* only | 25 (32.9) |
| *T. brucei* and *T. congo* | 10 (13.2) |
| *T. brucei* and *T. vivax* | 2 (2.6) |
| *T. congo* and *T. vivax* | 4 (5.3) |
| *T. brucei* and *T. congo* and *T. vivax* | 13 (17.1) |
